# In_3_SbTe_2_ as a programmable nanophotonics material platform for the infrared

**DOI:** 10.1038/s41467-021-21175-7

**Published:** 2021-02-10

**Authors:** Andreas Heßler, Sophia Wahl, Till Leuteritz, Antonios Antonopoulos, Christina Stergianou, Carl-Friedrich Schön, Lukas Naumann, Niklas Eicker, Martin Lewin, Tobias W. W. Maß, Matthias Wuttig, Stefan Linden, Thomas Taubner

**Affiliations:** 1 Institute of Physics (IA), RWTH Aachen University, Aachen, Germany; 2 Physikalisches Institut, University of Bonn, Bonn, Germany

**Keywords:** Nanophotonics and plasmonics, Metamaterials

## Abstract

The high dielectric optical contrast between the amorphous and crystalline structural phases of non-volatile phase-change materials (PCMs) provides a promising route towards tuneable nanophotonic devices. Here, we employ the next-generation PCM In_3_SbTe_2_ (IST) whose optical properties change from dielectric to metallic upon crystallization in the whole infrared spectral range. This distinguishes IST as a switchable infrared plasmonic PCM and enables a programmable nanophotonics material platform. We show how resonant metallic nanostructures can be directly written, modified and erased on and below the meta-atom level in an IST thin film by a pulsed switching laser, facilitating direct laser writing lithography without need for cumbersome multi-step nanofabrication. With this technology, we demonstrate large resonance shifts of nanoantennas of more than 4 µm, a tuneable mid-infrared absorber with nearly 90% absorptance as well as screening and nanoscale “soldering” of metallic nanoantennas. Our concepts can empower improved designs of programmable nanophotonic devices for telecommunications, (bio)sensing and infrared optics, e.g. programmable infrared detectors, emitters and reconfigurable holograms.

## Abstract (web summary)

Here, the authors introduce In3SbTe2 (IST) as a programmable material platform for plasmonics and nanophotonics in the infrared. They demonstrate direct optical writing, modifying and erasing of metallic crystalline IST nanoantennas, tuning their resonances, as well as nanoscale screening and soldering.

## Introduction

Many recent technological advances in key areas like telecommunication, lighting, health care, and solar energy harvesting have benefited from increasing control over light. Confining light waves to ever-smaller length scales and thus overcoming Abbe’s diffraction limit enables ultracompact optical components^1^. Plasmonics achieves this goal by employing evanescent electromagnetic modes supported by metallic nanostructures to concentrate light fields to deeply subwavelength regions^2,3^. Arranging plasmonic nanoantennas (“meta-atoms”) in subwavelength assemblies in so-called metasurfaces allows for realizing ultrathin optical devices with exciting functionalities like beam steering^4^, lensing^5,6^, and holography^7,8^. While these functionalities are originally fixed upon fabrication, they can be rendered active by multiple means including antenna geometry change by mechanical deformation^9–11^, charge carrier tuning by electric biasing^12,13^, and inducing an insulator-metal transition in VO_2_ by heating^14–17^. All of these tuning mechanisms are volatile, i.e., they only persist as long as an external stimulus is provided. In contrast, phase-change materials (PCMs) offer non-volatile tuning. Because of the huge property contrast between their amorphous and crystalline phases resulting from a unique bonding mechanism^18–21^, they enable exciting tuneable functionalities like waveguiding^22–24^, chemical sensing^25^, light detection^26^, and emission^27^, as well as lensing^28^. This makes them prime candidates for non-volatile nanophotonic applications such as integrated optical memories, color displays, or active metasurfaces^1,29^. The resonance wavelength of antennas in metasurfaces is approximately proportional to the effective refractive index of the antennas’ dielectric environment and size. So far, PCMs have been used in active metasurfaces^29,30^ to change the effective refractive index of the dielectric environment, leading to significant resonance shifts^31,32^. While PCMs have been successfully used previously to directly optically write dielectric antennas in thin PCM films^33–35^, the plasmonic properties of typical PCMs have only recently been reported^36,37^. However, they are limited to the near-infrared spectral range by the materials’ band gap, leaving the full infrared spectral range unexplored, up to now. By employing the next-generation non-volatile plasmonic PCM In_3_SbTe_2_ (IST)^38,39^, we tap here into this unfulfilled potential for PCMs for infrared plasmonics. IST is a semiconductor in the amorphous phase (aIST) but becomes metallic with negative permittivity in the whole-infrared range in the crystalline phase (cIST)^40^. Compared with other alternative plasmonic materials^41,42^, IST’s optical properties can be optically and locally switched. Thus, we propose a programmable nanophotonics material platform in the infrared based on it.

After comparing IST with common PCMs, we show how to write and erase plasmonic antenna arrays by optical switching on the single meta-atom level. Additionally, we demonstrate flexible antenna reconfiguration on the sub-meta-atom level by elongating, shortening and cutting individual rod antennas in half, causing significant resonance shifts. Next, we demonstrate optically tuneable, frequency-selective perfect absorbers based on cIST. Finally, we demonstrate how IST can be used together with conventional, prepatterned plasmonic metasurfaces to either screen individual antennas from incident light or to “solder” them together. Our concepts could lead towards fast and simple fabrication of plasmonic metasurfaces. Moreover, they can empower programmable functionalities of plasmonic devices for infrared optics, sensing and telecommunications. One could devise, for example, real-time (bio)sensing, in-situ pixel reprogramming of infrared detectors and displays, tuneable localized optical heating, and delicate post-fabrication metasurface adjustments to correct fabrication errors or minutely adapt the functionality (e.g., realizing ultracompact adaptive optics^43^).

## Results

### The phase-change material IST

**Fig. 1:**
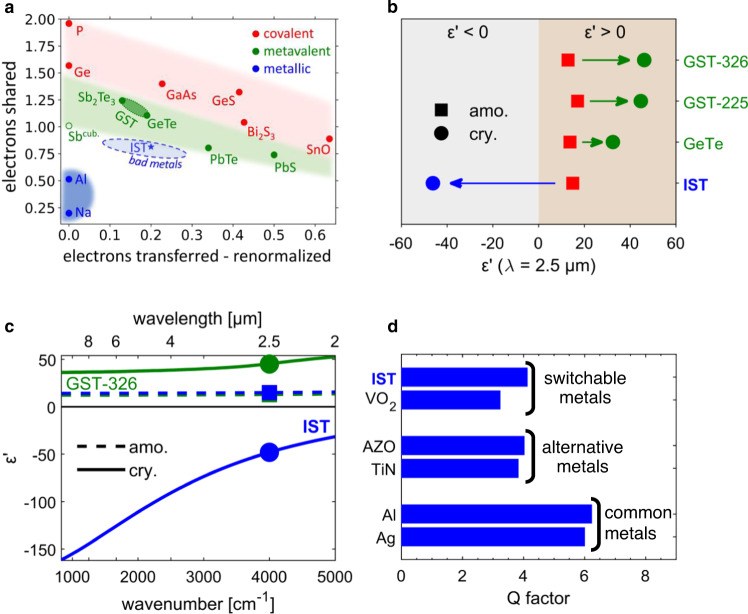
Optical properties of In_3_SbTe_2_ (IST). **a** Zoomed-in map describing bonding in solids. It is spanned by the renormalized electron transfer between adjacent atoms obtained after division by the formal oxidation state and the sharing of electrons between them. Common crystalline PCMs have metavalent bonds (green shaded area) and metals have largely delocalized free electrons (blue shaded area). IST is part of the material class “bad metals”: Its conductivity is significantly higher than that of other PCMs but smaller than that of metals. **b** The real part of the permittivity *ε*’ of IST (blue) in the infrared at 2.5 µm changes sign upon phase change, unlike common PCMs like GeSbTe compounds^49^. **c** Real part of the electric permittivity in the infrared of the crystalline and amorphous phases of GST-326 (green) and IST (blue), respectively, shown as a function of wavenumber. The data points from **b** are marked. **d** Calculated Q factor of rod antennas in free space consisting of the indicated materials. The antennas are 1-µm long, 0.4-µm wide and arranged in a square matrix with 2-µm period. IST as an antenna material is superior to VO_2_, TiN or AZO regarding the resonance quality.

PCMs are characterized by a significant change of their optical properties upon crystallization, which has been attributed to a change in bonding mechanism. Amorphous phase-change materials show the characteristic features of covalently bonded materials (moderate optical dielectric constant ε_∞_, low electrical conductivity and an atomic arrangement compatible with the 8-N rule^20^) like Phosphor and GaAs. Covalently bonded materials share about two electrons between adjacent atoms and are therefore located in the red shaded region of the bonding map^44^ in Fig. 1a (for a more detailed map see Supplementary Fig. 1 and its description). Crystalline phase-change materials such as GeTe, Sb_2_Te_3_ and Ge_2_Sb_2_Te_5_ (also called “incipient metals”^20^) on the contrary are characterized by an unconventional bonding mechanism coined metavalent bonding^18,21,45^ where about one electron is shared between adjacent atoms. Hence, ordinary phase-change materials like GeSbTe (GST) compounds are all located in the green shaded region of the map (see e.g., the denoted area between GeTe and Sb_2_Te_3_).

Remarkably now, crystalline IST has even less electrons shared between adjacent atoms^40^ and the electrons are significantly delocalized, more akin to a metal (blue shaded region in the map). The conductivity of IST at about 10^4^ S/cm^46^ is correspondingly also about 10–1000 times larger than that of crystalline GST^47^. This metal-like conductivity in the crystalline state leads to a very different change of optical properties upon crystallization and the potential to exploit extraordinary functionalities, as will be shown below. While GST compounds can be classified as “incipient metals”, IST belongs to the material class of non-volatile “bad metals”^48^, which form a bridge between the common PCMs and metals. Here, we reveal the opportunities offered by this interesting material class and IST especially for programmable infrared nanophotonics. To emphasize this potential, we denote IST as a plasmonic PCM.

The delocalization of electrons in the metavalent bond results in an increased polarizability and thus larger permittivity of common PCMs in their crystalline phase than in their covalently bound amorphous phase. This significant difference in permittivity can also be observed in Fig. 1b, where the real part of the permittivity *ε*‘ at 2.5 µm is plotted for several PCMs^49^ (see Supplementary Fig. 3 for more detailed permittivity plots). PCMs like GST compounds have a real permittivity *ε*‘ ≈ 10–20 in the infrared spectral range in their amorphous structural phase, with a negligible imaginary part. Upon crystallization, *ε*‘ increases by a factor of about two because of the change in bonding. These PCMs are semiconductors in both phases^50^ and the real part of their permittivity stays positive, *ε*‘ > 0, in this spectral region.

However, for crystalline IST the case is different: Because the electrons are delocalized similar to free electrons in a metal, IST changes from a semiconductor to a (bad) metal^51^ upon crystallization, exhibiting a change of the sign of the real part of the permittivity *ε*‘ from positive to negative (blue point in Fig. 1b). In Fig. 1c, the Drude-like permittivity of crystalline IST (blue) is evident (zero-transition at about 900 nm, see Supplementary Fig. 2) when compared to the permittivity of Ge_3_Sb_2_Te_6_ (GST-326).

The marked difference of optical properties in the crystalline phase between IST and other PCMs gives access to different antenna resonance tuning mechanisms. Instead of changing the refractive index Δ*n* of an antenna’s dielectric surrounding, IST can be used to directly change the antenna size by Δ*l* or even totally change the antenna shape itself. While the large refractive index change has been shown to be a powerful tool, it is limited by the naturally occurring refractive indices of PCMs (as mentioned, they are usually about twice as large in the crystalline phase than in the amorphous phase). In addition, the antenna is often not completely surrounded with PCM but, e.g., lies on a substrate, effectively lowering the influence of the PCM’s refractive index change. In contrast, there is almost no limit to the antenna reconfigurations that can be facilitated by IST: Even subdiffraction switching becomes possible when combining crystallizing and amorphizing pulses. Thus, the accessible degrees of freedom for designing infrared properties of antennas significantly exceeds that available with other PCMs.

As exploited by previous studies^36,37^, other PCMs, like GeSbTe compounds, usually also have a small spectral window (mostly in the VIS-NIR) where *ε*‘ < 0 due to interband transitions at energies larger than the band gap energy. Because the charge carriers are not sufficiently delocalized, however, their permittivity does not follow the typical form of the Drude model and stays close to zero there. As a measure for a material’s plasmonic performance in nanoresonators^52^, in Fig. 1d we calculated the Q factors of rod antennas in free space made from different materials (see Supplementary Fig. 4 for the optical properties). The Q factor is defined as Q = *ν*/FWHM, where *ν* is the resonance frequency. Common metals like Al and Ag have Q factors of about 6. It is noteworthy that Al has a slightly larger Q factor compared to Ag due to its smaller skin depth in the infrared (7.7 nm for Al and 11 nm for Ag at a wavelength of 2.5 µm). The Q factor for IST is about 4, which is significantly larger than that of VO_2_ and still slightly larger than that of TiN and AZO (alternative metals^41^). Not only for localized but also for propagating surface plasmon polaritons, IST has a superior figure of merit compared to GST in the interband transition region and VO_2_ (see Supplementary Fig. 5).

Active infrared metasurfaces based on PCMs can be switched between multiple operation states via heating^26,28^, or short electrical^53^ and optical pulses^33,34,54–57^. The electrical and optical switching of IST has been studied previously in the contexts of nanowires^58^ and data storage^39^, respectively. In the latter study, it was demonstrated that IST can be optically switched without phase separation on short time scales (about 50 ns). Optical addressing has recently been used with GeSbTe alloys to fabricate thin-film dielectric metasurfaces^33,34^ and to program individual meta-atoms in prepatterned PCM-incorporating metallic^55^ and dielectric metasurfaces^56^. Going one step further, it is now possible to directly write and erase metallic elements on and below the meta-atom level in a metasurface containing a thin IST film by nanosecond laser pulses. Compared to conventional lithography, no cumbersome additional steps like lift-off or etching are required and the whole metasurface can be erased and rewritten fast and easily at any time. This vastly expands the possibilities of how PCMs can be employed in active metasurfaces.

### Optical writing and erasing of plasmonic nanoantennas

**Fig. 2:**
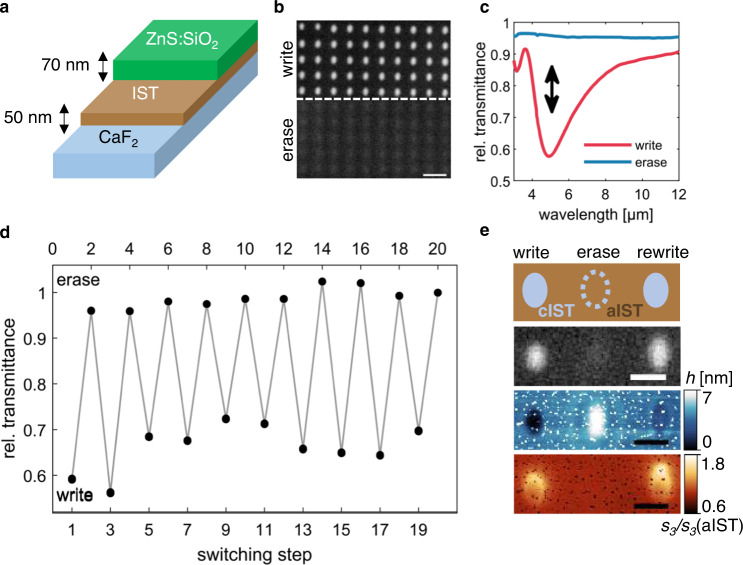
Write and erase. **a** Sketch of the sample layer stack. **b** Light micrograph (scale bar 3 µm) of a cIST nanoantenna array written (top) and erased (bottom) by optical switching. **c** FTIR transmittance measurements of the written and erased arrays from **b**. **d** Relative transmittance at 4.66 µm (resonance minimum) for 20 switching steps. **e** Sketch, light microscope, AFM (height *h*) and s-SNOM (*λ* = 10.57 µm, see Methods) images of written, erased and rewritten nanoantennas (scale bar is 1 µm).

As a platform for the following applications, we investigated how to write and erase plasmonic nanostructures on the meta-atom level in a thin film of 50-nm thick aIST on a CaF_2_ substrate (see Fig. 2). To prevent oxidization and ablation, the IST layer is capped with a 70-nm thick layer of (ZnS)_80_:(SiO_2_)_20_ (see Fig. 2a). A light micrograph of an optically written cIST nanoantenna array is shown in Fig. 2b. A pulsed laser with a wavelength of 660 nm was used for switching (see Methods). The bottom half of the array is replaced with the image of the erased array, where the cIST of each antenna was reamorphized. As shown, only a faint shadow of the antennas is left after erasing. The measured transmittance spectra of the written and erased arrays are shown in Fig. 2c. A clear resonance dip at *λ* = 4.66 µm is visible in the written state (red), whereas the resonance is completely gone in the erased state and almost unity transmittance is obtained (blue). To demonstrate the performance over several switching cycles, the transmittance at *λ* = 4.66 µm is plotted for the first 20 switching steps in Fig. 2d. It consistently switches between about 0.65 in the written state (bottom axis) and about 1 in the erased state (top axis). Finally, the written, erased and rewritten cIST nanoantennas are characterized by several microscopy methods (see Fig. 2e): light microscopy (LM), atomic force microscopy (AFM), and scattering-type near-field optical microscopy (s-SNOM, see Methods). The top row contains a sketch of the three switching states. Below that in the LM image, the written and rewritten cIST antennas are distinguishable as bright areas, whereas the erased antenna is not visible and appears as dark as the rest of the aIST film. The cIST areas appear bright because the real part of its permittivity *ε*‘ < 0 in the visible spectral range (because of interband transitions), which results in high reflectance in the light microscope. In contrast, aIST has *ε*‘ > 0 in the visible spectral range and thus reflects much less light. In the AFM topography image, however, all three cases can be discerned. The written and rewritten cIST antennas are visible as depressions in the aIST film (decreased density of crystalline PCMs^59^), while the erased antenna shows a slight elevation in topography (microbump formation^60^). With s-SNOM, it is possible to measure the local infrared optical properties of the submicrometer crystalline areas with a resolution that is well below the diffraction limit^61^. The measured near-field optical amplitude signal s_3_ at a wavelength of *λ* = 10.57 µm, referenced to the amplitude signal s_3_(aIST) of the aIST, is shown in the bottom row of Fig. 2e. Again, only the written and rewritten antennas, i.e., the cIST areas, are visible as bright features. The erased antenna shows the same optical contrast as the surrounding aIST film, confirming the successful reamorphization.

### Optical antenna resonance control

**Fig. 3:**
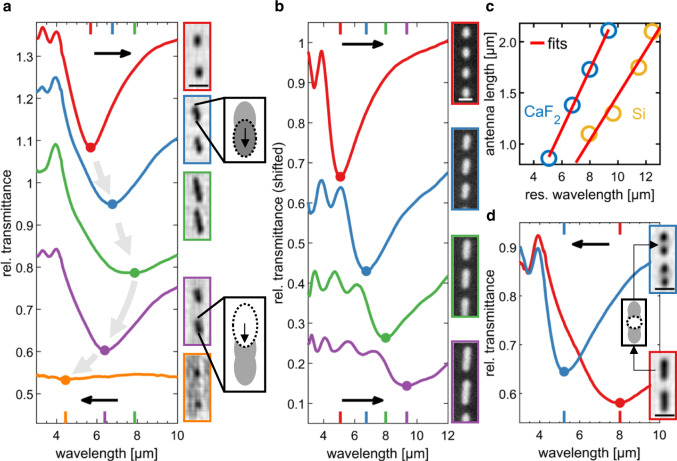
Optical antenna resonance control. **a** Measured transmittance spectra of nanorod antenna arrays of different length (shifted on *y*-axis for clarity). Light micrographs (transmittance mode) are shown on the right. The length was successively increased/decreased via crystallization/reamorphization at one antenna end (see sketches). **b** Measured transmittance spectra of nanorod antenna arrays of different length *l* and adjusted period (shifted on *y*-axis for clarity). Light microscope images of the antennas are depicted on the right. **c** Plot of the antenna length *l* against the measured resonance wavelength. Blue data points are for antennas on a CaF_2_ substrate, orange data points for antennas on a Si substrate. The red lines are linear fits to the two data sets and show good agreement with the data. **d** Measured transmittance spectra of long nanoantenna arrays (red) which were then optically cut in half (see sketch) by reamorphization (blue). Corresponding light micrographs are depicted on the right. All scale bars are 2 µm.

After establishing the viable writing and erasing of metallic nanostructures in an IST thin film on the meta-atom level, we performed first proof-of-principle experiments (see Fig. 3) to demonstrate the optical modification of infrared plasmonic antennas on the sub-meta-atom level. This effectively lays the foundation for sub-pixel reconfiguration and pixel-by-pixel programming in plasmonic metasurfaces (1 pixel = 1 meta-atom).

We employed the same layer stack as in Fig. 2a. Rod antennas were arranged in a 20 × 20 µm^2^ antenna array. After the initial writing, the antenna lengths were subsequently changed by switching the IST at the antenna tips. Figure 3a depicts the measured infrared transmittance spectra of the corresponding antenna arrays (see light microscope images on the right). In each spectrum, a minimum at the resonance wavelength (electric dipole resonance) can be observed as well as smaller features at shorter wavelengths originating from the antenna grating orders. The first antenna resonance lies at 5.7 µm (red). By crystallizing the IST at the antenna tips (see dark gray, dotted ellipse in the top sketch in Fig. 3a), the antennas are elongated and the resonance then gradually redshifts up to a wavelength of 7.9 µm. Next, the antennas were shortened by reamorphizing the IST at the antenna tips (see white, dotted ellipse in the bottom sketch in Fig. 3a). This led to a gradual blueshift from 7.9 µm down to 4.4 µm, totalling a complete tuning range of Δ*λ* = 3.5 µm.

Remarkably, the antennas can be made even shorter via reamorphization than the initial cIST antennas. This results in a significantly shorter resonance wavelength: 4.4 µm instead of 5.7 µm. By overlapping slightly shifted (diffraction-limited) crystallizing and reamorphizing laser pulses, it becomes possible to achieve optically switched structures on a scale below the diffraction limit of the VIS switching laser.

The longest antennas in Fig. 3a (green framed light micrograph) are already almost touching and cannot be elongated further. Longer antennas require a larger array period. Moreover, the amplitude for the shortest antennas is dramatically reduced due to their reduced area-density and cross-section. With our proposed material platform based on IST, it is now possible to completely rearrange the antennas in the array in addition to changing the antenna lengths (see Supplementary Movie 1). Figure 3b displays measured transmittance spectra and light micrographs of corresponding IST antenna arrays (see Methods), demonstrating a tuning range of Δ*λ* = 4.3 µm, which is more than 20% larger than the tuning range achieved for the static array periods in Fig. 3a. The experimental data are in good agreement with numerical simulations of infinite cIST antenna arrays of the same dimensions (see Supplementary Fig. 6).

The large total shift yields a large tuning figure of merit TFOM = Δ*λ*/FWHM ≈ 1.9. Here, the FWHM = 2.3 µm of the smallest antennas was considered in accordance with literature^31,62^. This is an exceptionally large TFOM value when compared with common GeSbTe compounds, which facilitate resonance shifts of Δ*λ* ≈ 1 µm and TFOM ≲ 1.2^32^. The resonance tuning with IST of Δ*λ* = 4.3 µm also surpasses other tuning concepts based on charge carrier density tuning (graphene^63^, doped semiconductors^64^) or on phase-transition materials like VO_2_^16^_,_ where Δ*λ* ≈ 1–2 µm.

The resonance wavelength (electric dipole) of classic metallic nanorod antennas follows the relation *λ*_res_ = *n*_eff_2*l* + *δ*, where *n*_eff_ is the effective refractive index of the antenna’s dielectric surrounding and *δ* is a constant offset parameter^65^. This linear relation is confirmed in Fig. 3c, where the antenna length is plotted against the resonance wavelength. The fit (red line) to the blue data points reveals the relation *λ*_res_ = 1.7·2*l* + 2.1 µm. Thus, the cIST antennas can be indeed described as plasmonic nanorod antennas. For substrates with a larger refractive index (e.g., about 3.4 for Si instead of 1.4 for CaF_2_), the effective refractive index *n*_eff_ of the antennas’ dielectric surrounding, and thus their resonance wavelength, is increased. Accordingly, by adjusting the substrate material, one can change the antenna lengths required for a certain spectral resonance position^66^ and optimize the resonance quality in that wavelength range (see Supplementary Fig. 7). As can be seen from the orange data points in Fig. 3c, the resonance wavelengths of cIST antennas on Silicon are about 2–3 µm larger than on CaF_2_ for the same antenna lengths and still follow a linear trend over the antenna length. The lower slope of the linear fit is due to Silicon’s larger refractive index. Moreover, the dipole antenna characteristics of the cIST antennas could be confirmed with numerical field simulations of the electric, magnetic and current density fields. Compared to gold antennas of the same dimensions, the field distributions are strikingly similar (see Supplementary Fig. 8).

Modifying cIST nanoantennas is not limited to adding or removing cIST at the antenna ends. In Fig. 3d, it is demonstrated that one can also directly access and modify the interior of individual antennas (meta-atoms). As depicted in the sketch (white dotted ellipse), the long nanorod antennas (red frame) were “cut through” in the middle by a reamorphizing pulse, resulting in two smaller rod antennas for each previous long antenna (blue frame). The measured transmittance spectra also reflect this with a blueshift of the resonance by 2.7 µm from 8.2 µm to 5.5 µm. This is only exemplary for the freedom given for design and modification of nanostructures in our introduced platform. One can switch the IST differently in each individual meta-atom and for instance write a metasurface where the antenna lengths increase along the array (see Supplementary Fig. 9a). Going beyond the modification of simple rod antennas, one can also write freeform shapes (see Supplementary Fig. 9b), as well as more complex resonator structures like split ring resonators by combining writing and erasing laser pulses (see Supplementary Fig. 10).

The creation of more complex structures also allows to access different resonance modes and to change between them: As shown, one can switch from a single electrical dipole to either two coupled electrical dipoles (cut nanorod) or to a magnetic dipole (split ring resonator). Higher-order modes like quadrupole resonances could also become attainable by reshaping or coupling of individual resonators.

### Optical writing of frequency-selective infrared absorbers

**Fig. 4:**
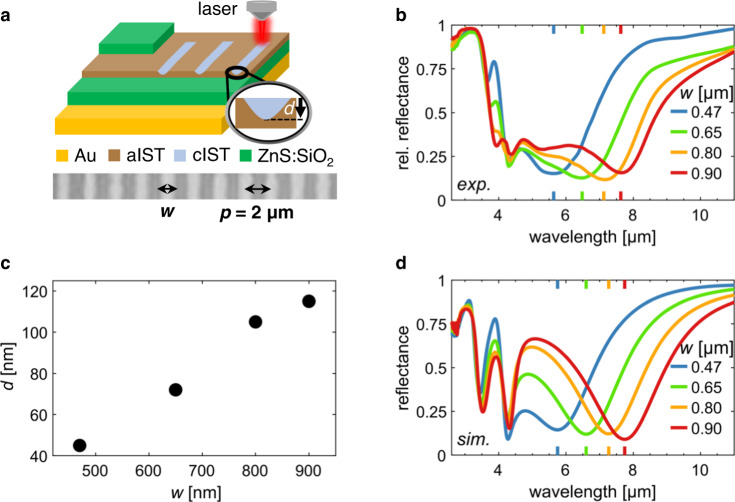
Optical writing of frequency-selective IR absorber. **a** Top: Sketch of the absorber layer stack and grating pattern structure. cIST bars of variable width *w* and period *p* can be written into the surface with the pulsed laser. The inset sketches the crystallization depth *d*. Bottom: Light microscope image excerpt of a cIST absorber, denoting the fixed period *p* = 2 µm and the bar width *w*. **b** Measured reflectance spectra of the IR absorbers with *p* = 2 µm configured to absorb at different spectral ranges by changing the bar width *w* from 0.47 to 0.9 µm. The magnetic resonance positions are marked with colored ticks. **c** Crystallization depth *d* of the simulated cIST bars vs. their width *w*. **d** Simulated reflectance spectra corresponding to **b**.

In another proof-of-principle experiment for optically written plasmonic IST metasurfaces, we fabricated a frequency-selective IR absorber (see Fig. 4). First, a 100-nm thick Au mirror is evaporated onto a Si substrate. On top of that, 230 nm (ZnS)_80_:(SiO_2_)_20_, 115 nm aIST, and 70 nm (ZnS)_80_:(SiO_2_)_20_ are deposited (Fig. 4a). According to the previously discussed concepts of optical writing, metallic cIST grating bars of variable width *w* and period *p* can be directly written into the unstructured layer stack (cf. Fig. 4b). The metallic bars and the Au bottom mirror form a resonant infrared absorber system. Because of the Au mirror, there is 0 transmittance and the difference of the reflectance *R* to unity equals the absorptance *A* = 1 − *R*. Moreover, absorption and emission are linked by Kirchhoff’s law of thermal radiation. Thus, good infrared absorbers are also good thermal emitters^67^. In our experiment, we set the period to *p* = 2 µm and varied *w* between 470 and 900 nm by adjusting the switching laser pulse duration. The incident infrared light was polarized perpendicularly to the bars. In the measured reflectance spectra (Fig. 4c), there is a reflectance minimum at wavelengths larger than 5 µm (magnetic resonance) and 2 significant minima at wavelengths smaller than 5 µm (thin-film resonances). The magnetic resonance is created by the oscillating charges in the cIST at the top and their mirror image in the Au at the bottom. When increasing the bar width *w* from 470 to 900 nm, one can now continuously spectrally tune the reflectance minimum associated with the magnetic resonance between about 5 and 8 µm. The minimum reflectance is always smaller than 15% and for *w* = 0.8 µm (green), it almost reaches down to 10%. This corresponds to a maximum absorptance of *A* ≈ 90 % and the tuning enables an absorptance modulation of up to 65%.

In Fig. 4c, the crystallization depth *d* is plotted against the bar width *w*. It is determined by a comparison of minimum reflectance between simulations and measurements. The simulated spectra (Fig. 4d) only fit well to the measured spectra if a vertical extent of the cIST bars is assumed that is smaller than the total aIST film thickness (inset in Fig. 4a). The existence and importance of this crystallization depth *d* for resonance tuning of GST-covered nanoantennas by optical switching has been demonstrated just recently^55,68^. Due to different thermal properties of the layers above and below the aIST layer, a temperature gradient arises from top to bottom. Because the temperature is highest at the top (air is a bad thermal conductor), crystallization starts there and then proceeds downwards. Experimentally, the different crystallization depths are realized by using increasing laser pulse durations for increasing cIST bar widths *w* (cf. Methods). The optical heating for longer times then allows for crystallization deeper into the aIST layer, resulting in larger crystallization depths. For the given absorber structure, the crystallization depth is an important parameter because it not only defines the total crystallized volume but also the distance between the plasmonic structures and the metallic back-mirror, which is directly related to their electromagnetic coupling and the resulting magnetic dipole moment. Only by optimizing the crystallization depth, the absorptance can be maximized to close to 100% for a given layer stack (see Supplementary Fig. 11).

Our frequency-selective IR absorber design has great potential for thermal imaging applications (see Supplementary Fig. 12) and leads the way towards optical programming of light absorbers^26^ for infrared color, both for detection and emission. This might for example be employed for programmable simultaneous thermal infrared invisibility and holographic illusion^69^.

### Nanoscale screening and soldering

So far, we have used unpatterend IST thin-films to directly optically write tuneable plasmonic antennas and active metasurfaces. In a different approach, one can also use IST to adjust prepatterned metallic antennas. Then, IST can either work like a nanoscale screen on the meta-atom level or like a “solder” for metallic nanostructures on the sub-meta-atom level. A similar concept, though designed for large-area tuning, has very recently also been demonstrated with the volatile phase-transition material VO_2_^70^.

**Fig. 5:**
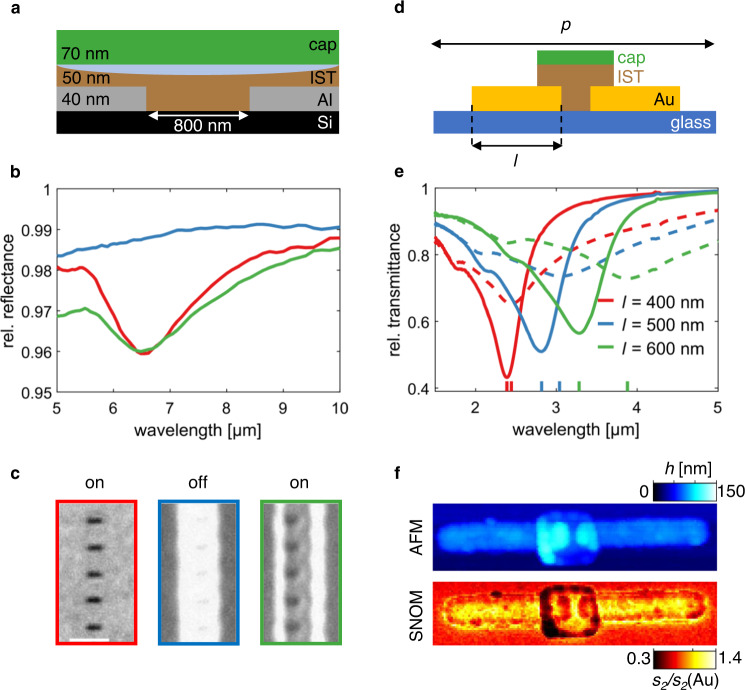
Nanoscale screening and soldering of nanoantennas with In_3_SbTe_2_. **a** Cross-section sketch of the full layer stack of an IST-covered Al slit antenna. The cIST (light blue) does not reach down completely through the aIST (brown) to the Al (silver). **b** FTIR reflectance spectra of slit arrays for the default on-state (red), the off-state (blue) and the restored on-state (green). **c** Light microscope image excerpts of the arrays measured in **b** (scale bar: 2 µm). **d** Cross-section sketch of the array unit cell with a gold dimer antenna on glass whose gap is bridged with an IST patch. **e** Measured transmittance spectra of the dimer antenna arrays with different antenna lengths *l* (400 nm, 500 nm, 600 nm) and period *p* = 2.4*l* for aIST (solid) and cIST (dashed). With cIST the dimers are soldered together and the resonance redshifts (see colored ticks at the bottom). **f** AFM (top) and s-SNOM (bottom) images of a gold dimer soldered together with cIST: In the s-SNOM image, the cIST patch has the same optical contrast as the Au antennas, verifying its metallic infrared optical properties.

Generally, each individual nanoantenna in an IST-coated metasurface can be permanently switched off (antennas not excited) and on (antennas excited) by optically switching the IST above the antennas. In the crystalline state, the cIST area acts like a mirror, which screens the antenna from the incident light and thus prevents antenna excitation. This is demonstrated here for the case of an IST-coated Al slit antenna array on a Si substrate (see Fig. 5a). In Fig. 5b, measured reflectance spectra are plotted for three different switching states: the default on-state (red), the off-state with cIST above the slits (blue) and the restored on-state with reamorphized IST above the slits (green). In the two on-states, there is a distinct reflectance minimum (slit resonance), whereas it is gone in the off-state. In Fig. 5c, the corresponding light micrographs are displayed. Please note that the resolution of these images is limited by diffraction and that the shape of the slits does not change because of the switching.

Until now, the switching of IST was performed optically and was therefore limited by diffraction of the red light of the switching laser. In case the IST needs to be switched at even smaller scales, it can be prepatterned with nanofabrication methods like electron beam lithography. In this way, ultrasmall IST switches can be strategically arranged on a metasurface and selectively toggled between dielectric and metallic optical properties at the sub-meta-atom level. As an application, we show how the two rods in a gold dimer antenna can be conductively connected by a small patch of IST positioned in the dimer gap (Fig. 5d). Thus, two neighbouring, separated nanoantennas can be turned into a single long nanoantenna. The results of such switching for a dimer antenna array with IST-bridged 100 nm dimer gaps are shown in the transmittance measurements in Fig. 5e. There, 50-nm thick IST and 10-nm thick ZnS:SiO_2_-capping are deposited exclusively at the 100 nm dimer gap of 150-nm wide, 30-nm thick Au antennas of different lengths *l* and period *p* = 2.4 *l*. The aIST was then crystallized by heating on a hotplate (see Methods). In the plotted transmittance spectra, the spectral redshift of the transmittance minimum (resonance) from aIST (solid lines) to cIST (dashed lines) patches is observable for each length *l*. This resonance shift resulting from the conductive connection between the dimer antennas becomes larger with increasing *l*. For *l* = 600 nm, it is about 600 nm large, which corresponds to a tuning figure of merit of about 0.7. Here, the resonance shift would originally be even larger, but the Au antenna lengths decreased after annealing in the oven (see Supplementary Fig. 13a). The kink in the spectra at smaller wavelengths results from grating resonances and thus stays at the same wavelength after switching the IST because the array periodicity is conserved even though the antenna lengths slightly decrease. The resonance shift will generally increase even more for longer antennas and larger gaps (cf. Supplementary Fig. 13b). The topography and near-field optical contrast (measured with s-SNOM at a wavelength of *λ* = 10.57 µm) of a dimer with 600 nm long antennas and a cIST patch are depicted in Fig. 5f. In both images, it is visible that the cIST patch in the dimer gap overlaps with both antennas. Notably, the near-field contrast of the cIST patch and the Au antennas is the same in the bottom image, which signifies similar optical properties. This underlines again the metallic infrared optical properties of crystalline IST.

Multiple potential nanoantenna geometries can now be encoded on a single metasurface with strategically positioned IST patches and then switched between by nanoscale soldering. On the one hand, this allows for minor adjustments, which is, e.g., important for compensating tight fabrication tolerances and for adaptive optics^43^. On the other hand, one can also realize major reconfigurations like a total change of functionality, e.g., between beam focusing and beam steering. Please note that the switching of prepatterned IST structures is not limited to optical laser pulses but can also be achieved via electrical voltage pulses^58^.

## Discussion

We introduced the next-generation, switchable, infrared plasmonic phase-change material In_3_SbTe_2_ (IST) as a material platform for programmable plasmonics and nanophotonics in the infrared. On the meta-atom level, we demonstrated direct writing and erasing of metallic crystalline IST (cIST) nanoantennas into a dielectric amorphous IST (aIST) thin film for 20 switching steps and characterized them by diffraction-limited far-field as well as super-resolution near-field measurement techniques. On the sub-meta-atom level, we demonstrate flexible antenna reconfiguration by elongating, shortening and cutting individual rod antennas in half, tuning the infrared plasmonic antenna resonances by more than 4 µm. Subsequently, a tuneable broad-band infrared absorber with absorptance of nearly 90%, a mid-infrared spectral tuning range of about 3 µm and an absorption amplitude tuning range of about 65% was shown. Nanoscale screening of prepatterned antennas with a thin IST film results in an on/off functionality, while strategically positioned IST patches allow for nanoscale “soldering” of individual meta-atoms.

These concepts are fundamentally impossible to achieve with common dielectric PCMs (e.g., GeSbTe alloys). In addition to a large resonance tuning, IST enables the creation of complex plasmonic structures with different resonance modes. The optical switching can be easily scaled up and parallelized (e.g., by laser interference lithography) towards large-area fabrication with high throughput. Our work thus lays the foundation for a programmable nanophotonics material platform for the infrared. It also holds great potential for application in integrated photonics, where, reconfigurable plasmonic components for, e.g., routing, waveguiding and filtering are now made possible by optically or electrically switched IST elements. The extension of our frequency-selective near-perfect IR absorber could lead towards real-time biosensing or the in-situ reprogramming of infrared detectors and displays working in absorption or emission mode.

A thin IST layer on top of negative permittivity materials that host surface waves such as polar crystals (phonon polariton) or doped semiconductors (plasmon polariton) could enable the writing of nanophotonic elements for polaritons like deeply subwavelength resonators or waveguides^17,34,71,72^. Composing dielectric metasurfaces^56,57,73–75^ out of amorphous IST could enable the switching from narrow Mie resonances to broad plasmonic resonances. Finally, the non-volatile switching of IST could be combined with the dynamic, volatile tuning methods offered by materials like VO_2_, leading to interesting functionalities like multilevel, intelligent switches^76,77^.

## Methods

### Sample fabrication

In_3_SbTe_2_ (IST) was deposited on the substrates by direct current magnetron sputtering, while (ZnS)_80_:(SiO_2_)_20_ was deposited with radio frequency magnetron sputtering. A LS 320 von Ardenne systems (base pressure 2 ∙ 10^−6^ mbar, 20 s.c.c.m. Ar flow, deposition rates 1 Å/s for IST and 0.3 Å/s for ZnS:SiO_2_) was operated in constant power mode (24 W for IST and 59 W for ZnS:SiO_2_) using stoichiometric targets of 99.99% purity. The Al slit antennas were fabricated by first thermally evaporating Aluminum (2 Å/s, 2 × 10^−5^ mbar base pressure) onto a Si substrate and then patterning the Al thin film with focused ion beam milling with a Helios NanoLab DualBeam 650 by FEI company (30 kV acceleration voltage, 83 pA beam current, 3 µs dwell time). The gold dimer antennas with IST in their gaps were fabricated by two-step electron beam lithography with PMMA as resist and Electra92 as conductive polymer. In the first step, gold was evaporated onto the patterned resist. After a lift-off process, the gold dimer antennas are left on the glass substrate. In the second step, the resist was only patterned in the gaps of the dimers. Onto this patterned resist, 50 nm IST and 10 nm (ZnS)_80_:(SiO_2_)_20_ were sputtered (see above). After another lift-off process, the finished gold dimers with IST in their gaps are left on the glass substrate.

### Switching of IST

The localized phase change of the IST thin-films was realized with an in-house-built laser setup^55^. A pulsed laser beam (wavelength *λ* = 660 nm) is focused through a 10-fold objective with a numerical aperture of 0.5 on the sample surface. The sample is placed on a Thorlabs NanoMax-TS (Max311/M) stage, which is movable in x-, y-, and z-direction and connected to a Thorlabs closed-loop piezo controller (BPC303). A custom program allows for the automated positioning of pulsed laser shots on the sample surface within 5 nm accuracy. By using long (~500 ns) and low-powered (~10 mW) laser pulses, an IST thin film can be locally crystallized by heating it above the glass transition temperature. The crystallized area has an elliptical shape, whose size can be tuned by changing the laser parameters (pulse power, pulse duration and number of pulses)^55^, see e.g., Supplementary Fig. 7b. By using short (~20 ns) and high-powered pulses (~300 mW), the crystalline PCM can be melted locally by heating it above the melting temperature, after which a rapid cooling (>10^9^ K/s) yields amorphization. The laser pulse parameters [power, duration, number of pulses] for Fig. 2 are [7.2 mW, 500 ns, 1000] for crystallization and [281 mW, 10 ns, 1] for reamorphization. For Fig. 3a, the laser pulse parameters are [8.2 mW, 500 ns, 1000] for crystallization and [205 mW, 10 ns, 1] and the array periods are [*p*_x_, *p*_y_] = [2.2 µm, 3.5 µm]. For Fig. 3b, the laser pulse parameters are [7.2 mW, 500 ns, 1000] and the antenna array dimensions [*l*, *p*_x_, *p*_y_] in µm are measured by light microscopy and given as [0.9, 2.1, 1.8], [1.4, 2.9, 2.2], [1.7, 3.8, 2.6] and [2.1, 4.7, 3.0]. For Fig. 3c, the laser pulse parameters are [8.2 mW, 500 ns, 1000] for crystallization and [182.3 mW, 10 ns, 1] for reamorphization the array periods are [*p*_x_, *p*_y_] = [2.2 µm, 3.5 µm]. In Fig. 4, the employed laser pulse power is 0.4 mW, the number of pulses is 500 and the pulse durations for the respective bar widths are: 250 ns for *w* = 0.47 µm, 310 ns for *w* = 0.65 µm, 340 ns for *w* = 0.8 µm, and 370 ns for *w* = 0.9 µm. The switching parameters for screening the slits in Fig. 5a, b are [53.5 mW, 1000 ns, 150] for crystallization and [394.5 mW, 10 ns, 1] for reamorphization. For crystallizing the IST patches in the dimer gaps in Fig. 5c–e, the sample was heated uniformly on in an oven at 250 °C for 30 min.

### FTIR spectroscopy

The Fourier transform infrared (FTIR) spectroscopy data were collected using a Bruker Vertex 70 interferometer coupled to a Bruker Hyperion 2000 microscope. The FTIR measurement parameters for the data from the Figures is as follows: 1000 scans with 16 cm^−1^ spectral resolution for Figs. 2, 3, and 5; 2000 scans with 32 cm^−1^ spectral resolution for Fig. 4. For the measurements from Figs. 3 and 4, ×15 Cassegrain objectives were used, for all the other measurements ×36 objectives. The area around the antenna arrays was excluded from the measurement with knife edge apertures and the polarization was along the long antenna axes in Figs. 2, 3, and 5e, and perpendicular to the grating bars and slits in Figs. 4 and 5b, respectively. For all measurements the free area on the sample next to the antenna arrays was used as a reference, except for those in Fig. 4, for which reflectance at an Au mirror was taken as reference.

### Scattering-type scanning near-field optical microscope (s-SNOM) measurements

Our s-SNOM system (Neaspec GmbH) is based on a tapping-mode AFM. The metallized AFM tip (radius ∼25 nm) vibrates with an amplitude of ~40 nm at a tapping frequency *Ω* ≈ 235–250 kHz. This oscillating tip is illuminated by light from a CO_2_ gas laser at 10.57 µm and the backscattered light from the tip is collected. To suppress background scattering, the detector signals are demodulated at a higher harmonic *nΩ*(*n* ≥ 2). By means of pseudo-heterodyne interferometric detection^78^, the near-field optical amplitude *s*_*n*_ and phase *φ*_*n*_ are obtained. For measurements in Fig. 2, the signals were referenced to the aIST film next to the crystalline spots and demodulated at 3*Ω*. For measurements in Fig. 5, the signals were referenced to the gold of the dimer antennas and demodulated at 2*Ω*.

### Simulations

Full-wave 3D simulations have been performed using a commercial solver, CST Studio Suite. Excitation by Floquet Mode Ports has been used to model the experiment setup and to calculate the spectra. Periodic boundary conditions have been used in the lateral directions. Spectra for angles of incidence between 8° and 24° were calculated and averaged according to the intensity distribution of the ×15 Cassegrain objective. The permittivity of IST is shown in Supplementary Fig. 2. For ZnS:SiO_2_, a constant refractive index of about 2.1 and for CaF_2_ a constant refractive index of 1.4 were assumed.

## Supplementary information

Supplementary Information

Description of Additional Supplementary Files

Supplementary Movie 1

## Data Availability

All data needed to evaluate the conclusions in the paper are present in the paper or the supplementary materials. The data that support the findings of this study are available from the corresponding author upon reasonable request.
