# Supplementary material for: In3SbTe2 as a programmable nanophotonics material platform for the infrared
Source: Nat Commun. 2021 Feb 10;12:924. doi: 10.1038/s41467-021-21175-7 (PMC7876017; doi:10.1038/s41467-021-21175-7)
Supplement: Supplementary file 1 — Supplementary Information [file 41467_2021_21175_MOESM1_ESM.pdf]

Supplementary Information

for

In_3SbTe_2 as a Programmable Nanophotonics Material Platform for the Infrared

Andreas Heßler[1,#], Sophia Wahl[1], Till Leuteritz[2], Antonios Antonopoulos[1], Christina Stergianou[1], Carl-Friedrich Schön[1], Lukas Naumann[2], Niklas Eicker[1], Martin Lewin[1], Tobias W. W. Maß[1], Matthias Wuttig[1], Stefan Linden[2], Thomas Taubner[1,]*

[1] Institute of Physics (IA), RWTH Aachen University, D-52056 Aachen, Germany

[2] Physikalisches Institut, University of Bonn, D-53115 Bonn, Germany

[#] Email: hessler@physik.rwth-aachen.de

[*] Email: taubner@physik.rwth-aachen.de

Content:

Supplementary Note 1: Classifying of In_3SbTe_2 within the bonding map

Supplementary Note 2: Comparing permittivities of In_3SbTe_2 (IST) and of $Ge_3Sb_2Te_6$ (GST)

Supplementary Note 3: Optical properties of several plasmonic materials

Supplementary Note 4: Plasmonic figure of merit

Supplementary Note 5: Simulations for optical antenna resonance control

Supplementary Note 6: Antenna resonance tuning on Si substrates

Supplementary Note 7: Simulated electromagnetic near-fields of cIST antennas

Supplementary Note 8: Demonstration of flexible optical switching

Supplementary Note 9: Crystallization depth in the IST absorber

Supplementary Note 10: Polarization-dependent infrared imaging with the IST absorber

Supplementary Note 11: Nanoscale soldering of nanoantennas

References

Appended: Supplementary Movie 1: Antenna array reconfiguration

Supplementary Note 1: Classifying of In_3SbTe_2 within the bonding map

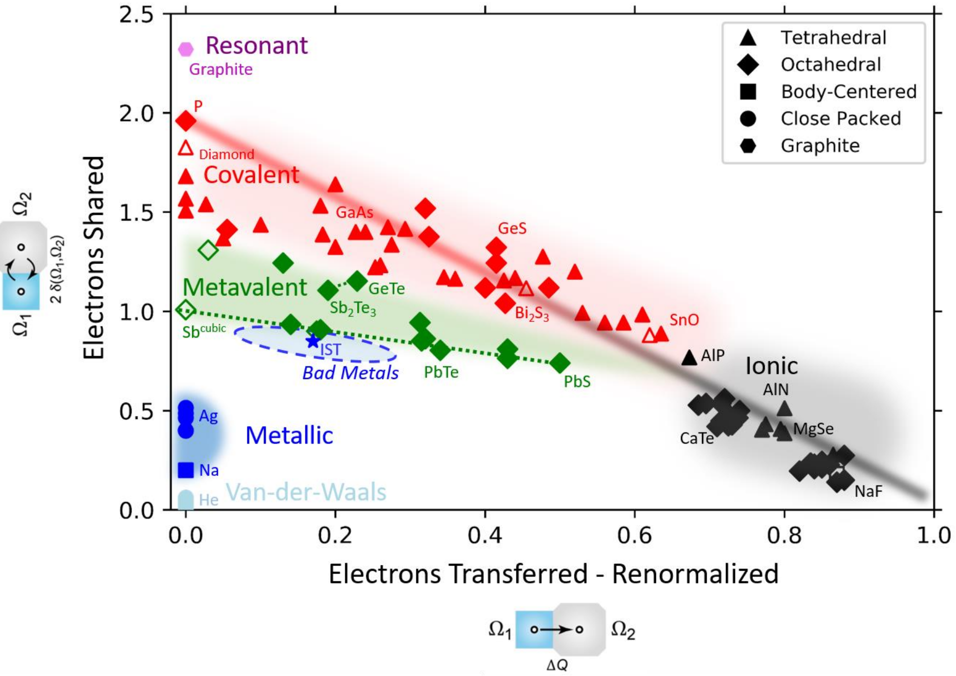

Supplementary Figure 1: 2-dimensional map describing bonding in solids. The coordinates of the maps are the renormalized electron transfer, obtained by dividing the electron transfer to/from an atom by the formal oxidation state, as well as the number of electrons shared between two adjacent atoms. This map separates different bonding mechanisms. Different markers (triangles, diamonds, squares, circles) are used to represent different atomic arrangements. Filled symbols represent thermodynamically stable phases, open symbols metastable phases. All materials on the green-dotted line assume an undistorted rocksalt structure with p-bands being half-filled. All compounds with distorted octahedral structures are located above this dotted line, exhibiting a larger amount of electrons shared. The blue ellipse below the green-dotted line designates the area where "bad metals" are located on the map.

PCMs are characterized by a significant change of their optical properties upon crystallization, which has been attributed to a change in bonding mechanism. For describing and quantifying the bonding in solids, employing the Quantum Theory of Atoms in Molecules (QTAIM) has led to significant progress[1]. In order to utilize this Quantum Chemical Topology scheme, a crystal is divided into non-overlapping domains corresponding to quantum atoms.[2] From these domains, in combination with the electron distribution within the crystal calculated by density functional theory, several parameters can be derived. One of them is the domain population, which characterizes the electron transfer from or to a basin. As the maximal charge transfer is limited by the formal oxidation state of an atom, it is reasonable to renormalize the total charge transfer by dividing it by the formal oxidation state, yielding the "Electrons Transferred – Renormalized" property. The second parameter is the delocalization index (DI), which is a metric to describe the degree of electron delocalization between two domains and can be interpreted as the covalent bond order[3,4]. Twice the delocalization index expresses the number of electrons shared between two atoms and is hence called "Electrons Shared".

Supplementary Figure 1 employs both these parameters, (the absolute value of) "Electrons Transferred – Renormalized" and "Electrons Shared", as the x- and y-axis of a 2D map for an extensive number of elemental and binary solids, as well as a limited amount of more complex compounds. Coloring the compounds by their respective type of chemical bonding,

it becomes apparent that this map separates materials according to their bonding mechanism. It also provides further support for the introduction of a novel bonding mechanism, called metavalent bonding.
The lower left corner is populated by the solids of noble gases (light blue), such as Ne and Ar. They are characterized by negligible charge transfer and very limited electron sharing. This behavior is typical for compounds held together by weak van der Waals forces. Ionic solids, e.g. NaCl and MgO, are located in the bottom right corner of the map (black), characterized by small electron sharing and pronounced electron transfer, in line with textbook knowledge. Covalently bonded compounds (red), like Si and GaAs, are characterized by pronounced electron sharing, and relatively low electron transfer, consistent with the classical concept of covalent bonding being based on shared electron pairs. By increasing the electron transfer, the electron shared value decreases and the covalent solids approach the ionic region and continuously transition from covalent to ionic bonding. Small to vanishing charge transfer in combination with a small number of shared electrons is characteristic for metals (blue), like Ag and Na[5]. The area of metavalently bonded materials (green) contains chalcogenides such as GeTe, PbSe, Sb_2Te_3, and $AgSbTe_2$ and can generally by described as the competition zone between localization and delocalization.[6] The classification into different bonding mechanisms, as well as a more detailed justification for metavalent bonding as an independent bonding type is based on discontinuous property transitions while crossing the border from one bonding type to another. It is discussed in more detail in Ref. 6.
For this publication, In_3SbTe_2 has been calculated and added to the map (ET=0.20, ES=0.82). It is located in the metallic region just below the metavalent area, where “bad metals” are found. Such “bad metals” are characterized by an electrical conductivity, which is about one or two orders of magnitude smaller than the electrical conductivity of Ag or Cu. They show an almost Drude-like optical reflectivity, albeit with lower absolute reflectance and a more pronounced frequency dependence.

An interactive version of the bonding map can be accessed at: materials-map.rwth-aachen.de

Supplementary Note 2: Comparing permittivities of In_3SbTe_2 (IST) and of $Ge_3Sb_2Te_6$ (GST)

In_3SbTe_2 (IST) and $Ge_3Sb_2Te_6$ (GST) have similar permittivities in their amorphous structural phase (Supplementary Figure 2a). While the imaginary parts (dashed) lines are almost completely identical, the real part (solid lines) is about 2-3 larger for IST than for GST. When comparing the permittivities of the crystalline structural phases, the situation changes significantly. For crystalline GST (cGST), the real part of the permittivity approximately doubles and the imaginary part increases, especially in the infrared spectral range for wavenumbers smaller than 5000 cm^{-1} (Supplementary Figure 2b). In contrast, for crystalline IST (cIST), the real part of the permittivity becomes negative and the imaginary part increases, following a Drude relation (Supplementary Figure 2c).

The permittivity of IST can be described according to the model proposed by Shportko et al..[7,8] The amorphous phase permittivity can be described by a Tauc-Lorentz oscillator model with the imaginary part:

$$Im(\varepsilon_{TL}(\omega)) = \frac{A}{\omega}\frac{\omega_0\gamma(\omega-\omega_g)^2}{(\omega^2-\omega_0^2)^2+\gamma^2\omega^2}\Theta(\omega-\omega_g), \tag{1}$$

where ω_0 is the resonance frequency of the oscillator, γ is the resonator damping, A is the resonator strength, ω_g is the band gap frequency and Θ is the Heaviside function. The real

part of the permittivity is then given by the Kramers-Kronig relations and an additional term ε_∞ which accounts for the polarizability in the higher frequency range.

For the crystalline phase permittivity, a Drude term must be added:

$$\varepsilon_{Drude}(\omega) = \frac{\omega_p^2}{\omega(\omega + i\gamma_D)}, \quad (2)$$

where ω_p is the plasma frequency and γ_D is the Drude damping.

The Tauc-Lorentz-Drude model parameters for amorphous and crystalline IST are summarized in Supplementary Table 1.

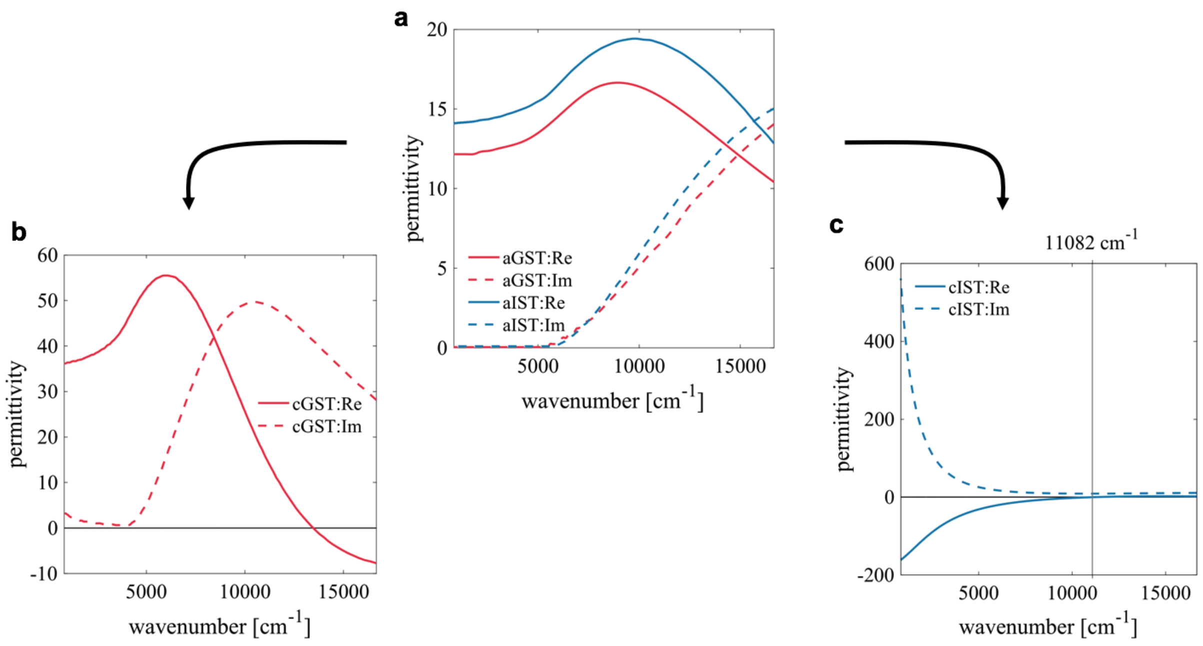

Supplementary Figure 2: Comparing dielectric functions of $Ge_3Sb_2Te_6$ (GST) and In_3SbTe_2 (IST). a) GST and IST have similar permittivities in their amorphous phase. **b)** For crystalline GST, the real part increases to about double the amorphous value, while the imaginary part is relatively small in the IR (<5000 cm^{-1}). c) In contrast, the permittivity of IST follows a Drude-relation, with the zero-transition at about 11080 cm^{-1} ≈ 900 nm.

Supplementary Table 1: Tauc-Lorentz-Drude model parameters for IST.

	amorphous IST	crystalline IST
A [Hz]	$13.0\cdot10^{16}$	$4.0\cdot10^{16}$
ω_0 [Hz]	$4.1\cdot10^{15}$	$4.1\cdot10^{15}$
ω_g [Hz]	$0.9\cdot10^{15}$	0
γ [Hz]	$5.2\cdot10^{15}$	$4.1\cdot10^{15}$
ω_p [Hz]	-	$7.0\cdot10^{15}$
γ_D [Hz]	-	$0.5\cdot10^{15}$
ε_∞	2	1.4

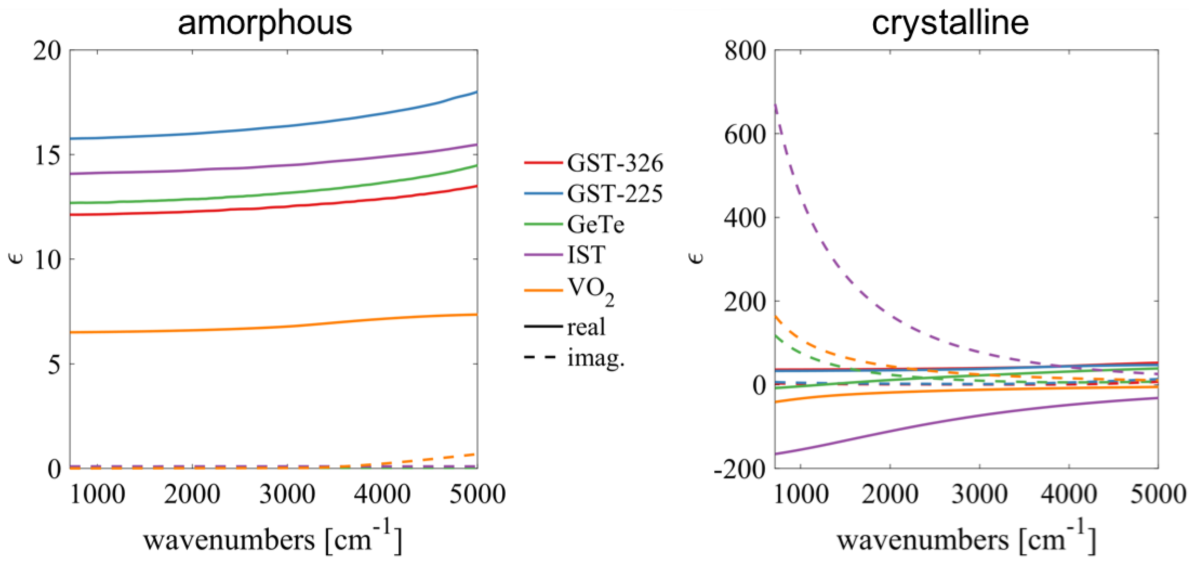

Supplementary Figure 3: Complex permittivities for materials from Figure 1 (main text). On the left, the permittivities for the amorphous phase of several PCMs as well as the semiconductor phase of VO_2 are plotted in the infrared spectral range. The solid lines are the real parts and the dashed lines the imaginary parts. On the right, similarly, the permittivities of the same materials are shown for the crystalline PCM phases and metallic phase of VO_2. VO_2 data was provided by Folland et al.[9].

Supplementary Note 3: Optical properties of several plasmonic materials

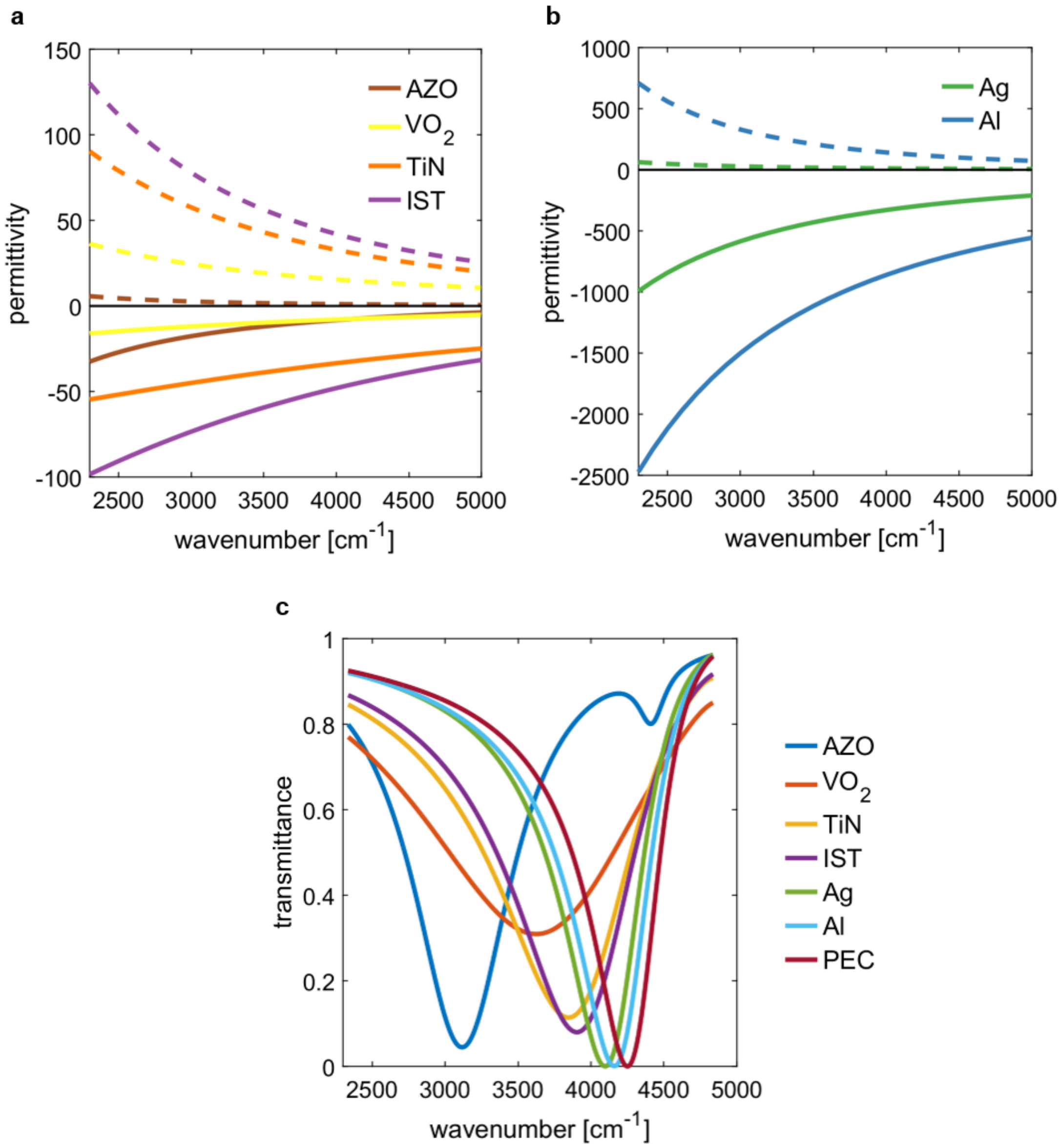

Supplementary Figure 4: Supplementary material for Figure 1d. **a,b)** Real (solid) and imaginary (dashed) parts of the permittivity of several materials in the mid infrared spectral range. **c)** Simulated transmittance spectra of rod antennas in free space, which are 1 µm long, 0.4 µm wide and arranged in a 2 µm period square array. The data for AZO[10], VO_2[9], TiN[11], Ag[12] and Al[12] were taken from literature.

Supplementary Figure 4 displays the permittivity of the plasmonic materials which are used in Figure 1d of the main text. They all show a Drude-like behavior with decreasing real parts and increasing imaginary parts for decreasing wavenumbers. One should make note of the different orders of magnitude of the permittivity between the alternative metals in the IR (Supplementary Figure 4a) and conventional metals (Supplementary Figure 4b). In Supplementary Figure 4c, transmittance spectra of rod antennas in free space made from these materials are shown. The antennas are 1 µm long, 0.4 µm wide and arranged in a square matrix with 2 µm period. From these spectra, the Q factors shown in Figure 1d of the main text were evaluated.

Supplementary Note 4: Plasmonic figure of merit

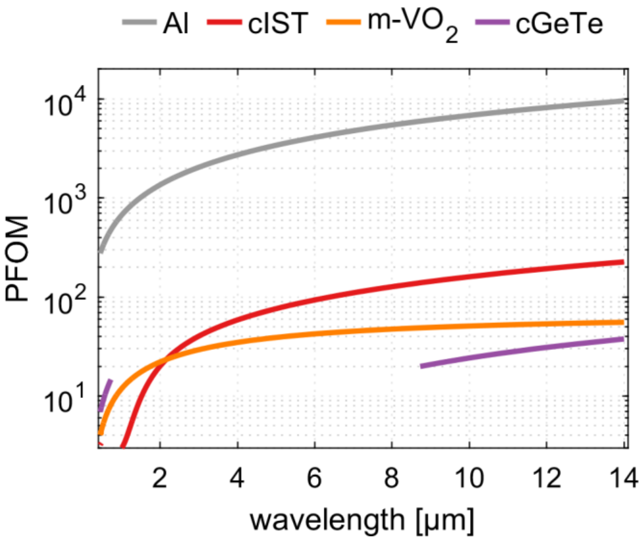

Supplementary Figure 5: Plasmonic figure of merit: Comparison of the plasmonic figure of merit (PFOM= $\mathrm{Re}(k_{SPP})/(2\pi\ \mathrm{Im}(k_{SPP}))$) of cIST with several other materials. While not as good as a metal (Al), IST is superior to crystalline GeTe (cGeTe) and metallic VO_2 (m-VO_2) throughout the whole infrared range.

Supplementary Figure 5 displays the plasmonic figure of merit (PFOM) for several plasmonic materials. It is defined as PFOM = $\mathrm{Re}(k_{SPP})/(2\pi\ \mathrm{Im}(k_{SPP}))$, where k_{SPP} is the surface plasmon polariton wavevector, and effectively compares the propagation length of the polariton to its wavelength. Notably, PFOM > 1000 in the infrared for metals like Al (grey line). While the PFOM for crystalline IST (red line) is almost 2 orders of magnitude below this at PFOM ≈ 100, it is still significantly larger that of the volatile transition materials VO_2 (orange line) and that of crystalline GeTe (violet line). The latter can only be calculated at very short or very long wavelengths because only there the real part of the permittivity is negative. In contrast, the real part of the permittivity of IST is negative for the whole infrared range.

For short wavelengths, the negative permittivity of crystalline GeTe and other GeSbTe compounds results from interband transitions instead of free electrons, whereas for IST it stems exclusively from free electrons (see main text). This results in a significantly less negative permittivity for the GeSbTe phase-change materials in the VIS/NIR spectral range and a much smaller PFOM < 20 (see also the work of Gholipour et al.[13]).

Supplementary Note 5: Simulations for optical antenna resonance control

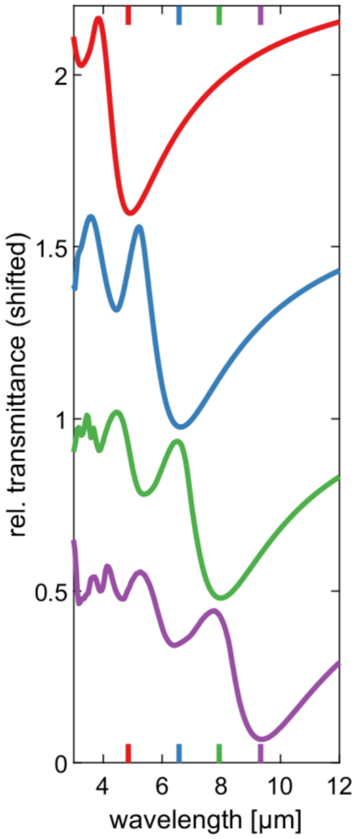

Supplementary Figure 6: Simulations for the optical antenna resonance control: Analogously to main text Figure 3b), transmittance spectra for antenna arrays of increasing length and period are displayed. The resonance minimum redshifts successively from top (red, small lengths and periods) to bottom (violet, large lengths and periods). The simulations are in good agreement with the experiment.

Supplementary Note 6: Antenna resonance tuning on Si substrates

As mentioned in the main text, a rod antenna resonance can be approximated by

$$\lambda_{res} = n_{\mathrm{eff}} 2l + \delta, \tag{3}$$

where n_{eff} is the effective refractive index of the antenna's dielectric surrounding, l is the antenna length and δ is a constant offset parameter.[14] The effective refractive index results from a weighted average of the refractive index of air (1), amorphous IST (~ 3.7) and the substrate material. In Figures 2 and 3 of the main text, the substrate material is CaF_2 which has a refractive index of about 1.4 in the infrared. This results in antenna resonances which can be tuned easily between about 4 and 10 μm. While the minimum resonance wavelength is limited by the diffraction limited resolution for the switching laser (λ = 660 nm), the maximum resonance wavelength is limited by the cIST inherent damping. For longer antennas, the damping increases. Thus, resonance wavelengths larger than 10 μm on CaF_2 come together with damped resonances. These resonance wavelengths can however easily be reached, as well, by substituting the CaF_2 substrate material with an optically denser material, for example Si. Silicon has a significantly larger refractive index (~ 3.4) than CaF_2, which leads to a larger effective refractive index n_{eff} in the above equation (3). Experiments to verify this were performed and the results are shown in Supplementary Figure 7. The layer stack is sketched in Supplementary Figure 7a. It is the same as in Figures 2 and 3 of the main text, except for the substrate material. Next, cIST antennas of varying length were optically written

into the aIST layer by using different laser pulse parameters (light micrographs in Supplmentary Figure 7b), as summarized in Supplementary Table 2. The resulting FTIR reflectance spectra are displayed in Supplementary Figure 7c. The measurements are normalized to aIST/Si next to the antenna arrays. The spectra are shifted with respect to the red curve by 0.07, 0.04 and 0.04, respectively. A pronounced reflectance peak can be seen in all the spectra. It stems from the cIST antenna resonance. Moreover, the resonance wavelength increases together with the antenna size, as expected. As a result, the resonance range was now shifted from 4-10 µm to 8-14 µm by changing the substrate material from CaF_2 ($n \approx 1.4$) to Si ($n \approx 3.4$). Further adjustments of the resonance tuning range can now easily be done by selecting the according substrate materials.

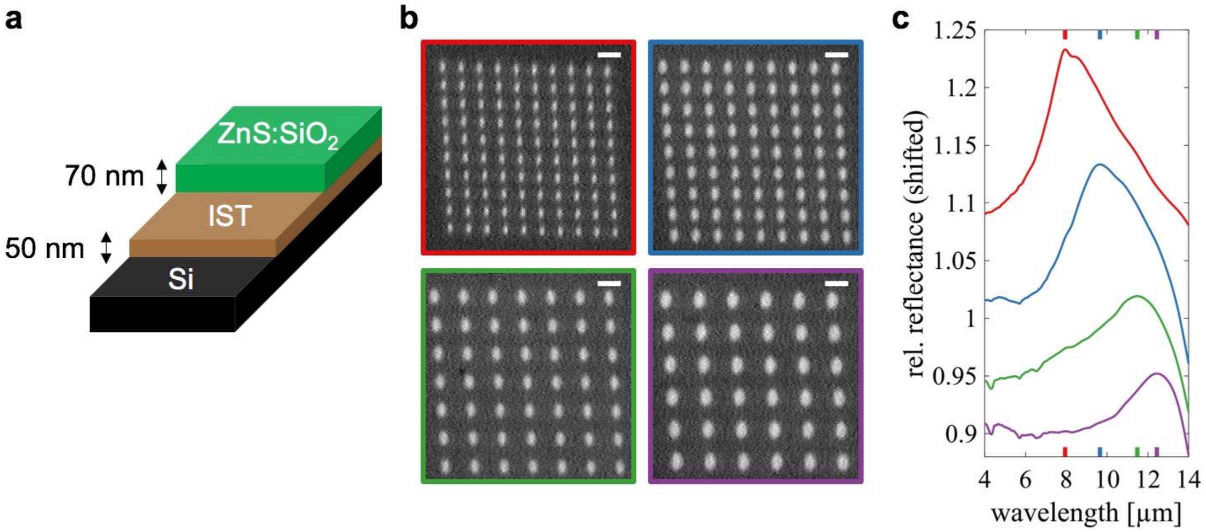

Supplementary Figure 7: Antenna resonance tuning on Si substrate. a) Sketch of the layer stack. **b)** Light micrographs of cIST antenna arrays with different antenna length *l*: 1.1 µm (red), 1.3 µm (blue), 1.75 µm (green) and 2.1 µm (violet). The scale bar is 3 µm. **c)** Measured reflectance spectra of the antenna arrays in b). An increasing redshift with increasing antenna length is apparent.

Supplementary Table 2: Laser pulse parameters for the cIST antennas in Supplementary Figure 7. All antennas were switched with 200 pulses during 1 second.

Antenna length [µm]	1.1	1.3	1.75	2.1
Pulse power [mW]	12.4	15.9	17.6	19.4
Pulse duration [µs]	3	1	1	1

Supplementary Note 7: Simulated electromagnetic near-fields of cIST antennas

When comparing crystalline IST to metallic antennas, it is instructive to have a look at the near-fields and current densities in such antennas. For this purpose, Supplementary Figure 8 displays numerical simulations of antennas with l = 1.54 µm from Figure 3 of the main text. In the top row, the antennas are made of cIST (like in the main text), and in the bottom row they are made of Au. The first column shows the normalized scattered electric field at resonance in a cross-section at half-antenna-height from the top. While the fields are larger for the Au antennas, the general field distribution is almost identical. A notable difference is the non-zero field inside of the antenna volume. This can be explained with the real part of the permittivity of cIST, which is much closer to zero than that of Au. Thus, the fields are not completely expelled from the antenna volume for cIST. When comparing the scattered

normalized magnetic field (second column) and the current density, similar comparisons can be made.
As a conclusion, the cIST antennas display very similar field distributions compared to the Au antennas. The decreased field amplitudes can be explained by the field penetration into the antenna volumes.

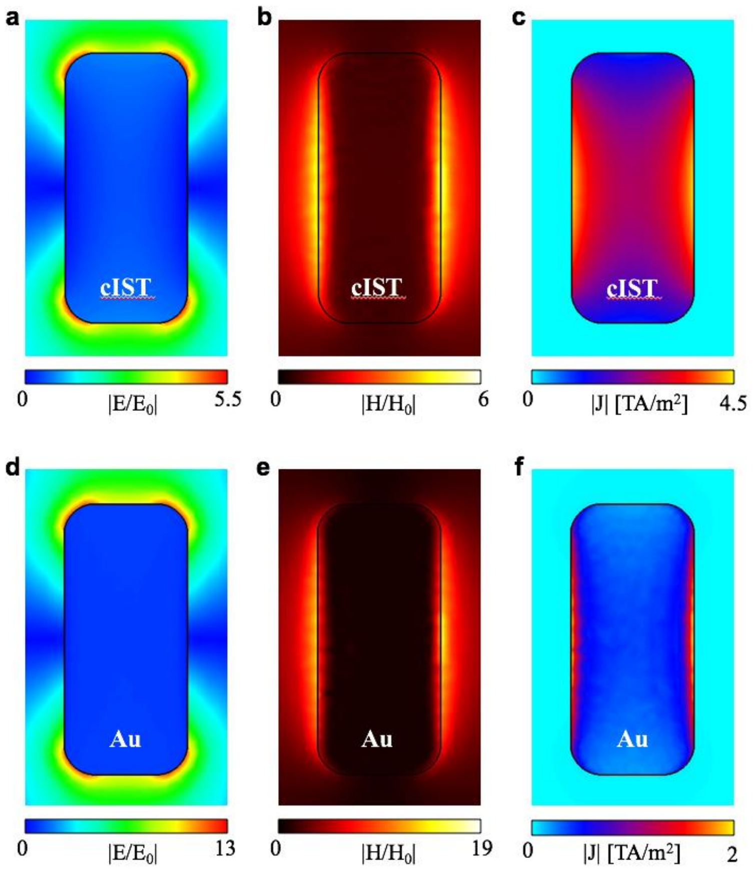

Supplementary Figure 8: Simulated electromagnetic near-fields of a cIST antenna. Comparing top-views on the cross-section at half IST-film thickness for antennas with l = 1.54 µm from Figure 3 (main text) at resonance for cIST- and Au-antennas: **a,d)** normalized scattered electric field amplitude, **b,e)** normalized scattered magnetic field amplitude and **c,f)** the current density. It is evident that the cIST antenna behaves in all cases like an Au-nanoantenna. The angle of incidence is 0° and the light is polarized along the long antenna axis.

Supplementary Note 8: Demonstration of flexible optical switching

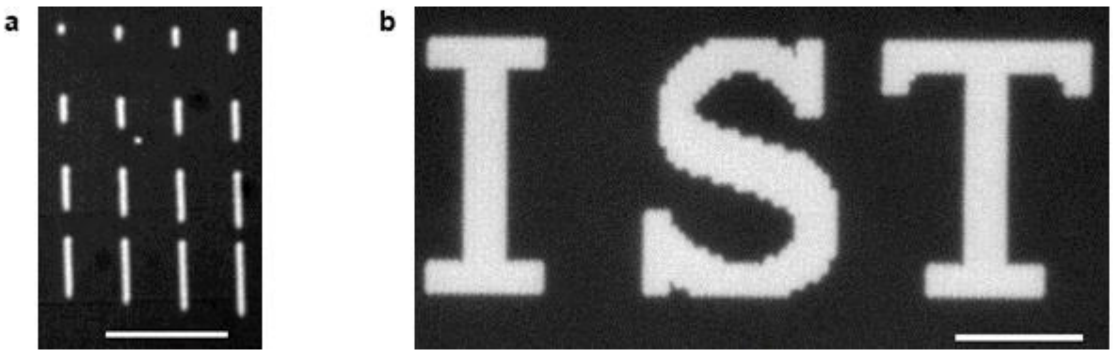

Supplementary Figure 9: Flexible optical switching. a) Light micrograph of a cIST rod antenna array with increasing antenna lengths along the array from the top left to the bottom right. The laser parameters are the same as for Figure 3a. b) Light micrograph of the cIST letters “IST” as an example

for freeform shapes written by optical switching. The laser parameters are [9.9 mW, 500 ns, 1000 pulses]. The scale bars are 10 µm long.

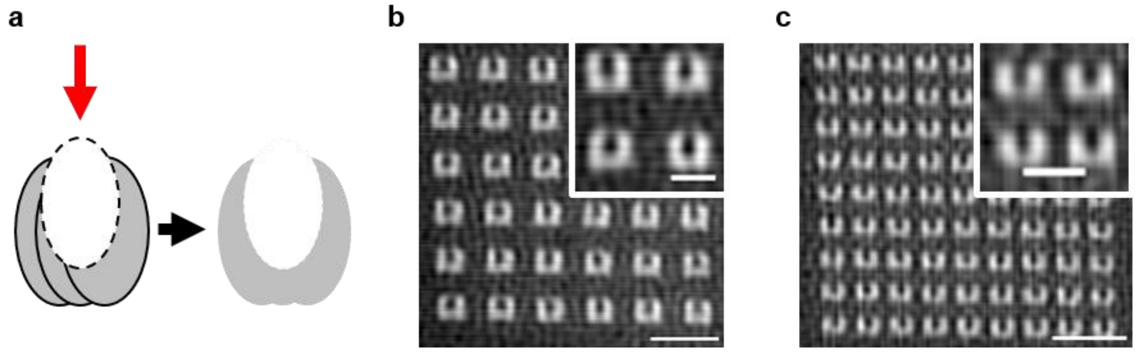

Supplementary Figure 10: Opitcal split ring resonator writing. a) Sketch of the working principle: By first combining multiple crystalline IST spots, a base is created. Next, a re-amorphizing pulse placed such on this base that the left-over crystalline part forms a U-shape. b, c) Light micrographys of optically written IST split ring resonators. The employed laser parameters are [8.2 mW, 500 ns, 1000 pulses] for crystallizing and [182.3 mW (b)/186.1 mW (c), 10 ns, 1 pulse] for re-amorphizing. The scale bars for the large images are 5 µm, those for the smaller insets are 2 µm.

Supplementary Note 9: Crystallization depth in the IST absorber

It is stated in the main text that a crystallization depth *d* has to be taken into account when modelling the cIST grating absorber. In Supplementary Figure 11a, the cross-section of the grating unit cell is sketched (same layer thicknesses as in Figure 4 of the main text). One could now naively assume that the cIST bar has a vertical rectangular cross-section and d = 115 nm. Based on TEM investigations of PCM thin films made of $Ge_3Sb_2Te_6$ which were optically switched with the same setup[15], the vertical cross-section is probably more elliptical than rectangular due to the gaussian intensity distribution of the switching laser beam. Moreover, there should be a laser parameter dependent crystallization depth, with larger *d* for longer pulse durations, i.e. cIST bar widths *w*. The laser parameters and resulting cIST bar widths are summarized in the Methods section of the main text and again in Supplementary Table 3. In Supplementary Figure 11b, the naïve, rectangular cross-section with full crystallization depth (dashed lines) is compared with the more likely elliptical cross-section with variable crystallization depth *d* (solid lines) from Figure 4 of the main text. The incident angle is set to 20° and the light is polarized perpendicular to the cIST bars. It can be seen that the reflectance minimum is generally at slightly larger wavelengths for the naïve cross-section. This is because the tapered-off sides of the bar in the elliptical cross-section slightly reduce the effective resonator length. Moreover, it becomes evident that for smaller bar widths, the resonance amplitude is significantly different. The naïve cross-section with full crystallization depth cannot reproduce the measured FTIR data from Figure 4 at all. Only when the crystallization depth is taken into account can the amplitude be matched to the experimental values. From the comparison of the elliptical cross-section simulations with the measurements, one can extract values for the crystallization depth (cf. Figure 4c in the main text). Similar reasoning has also been shown by Michel and Heßler et al. in the supporting information of their paper[15].

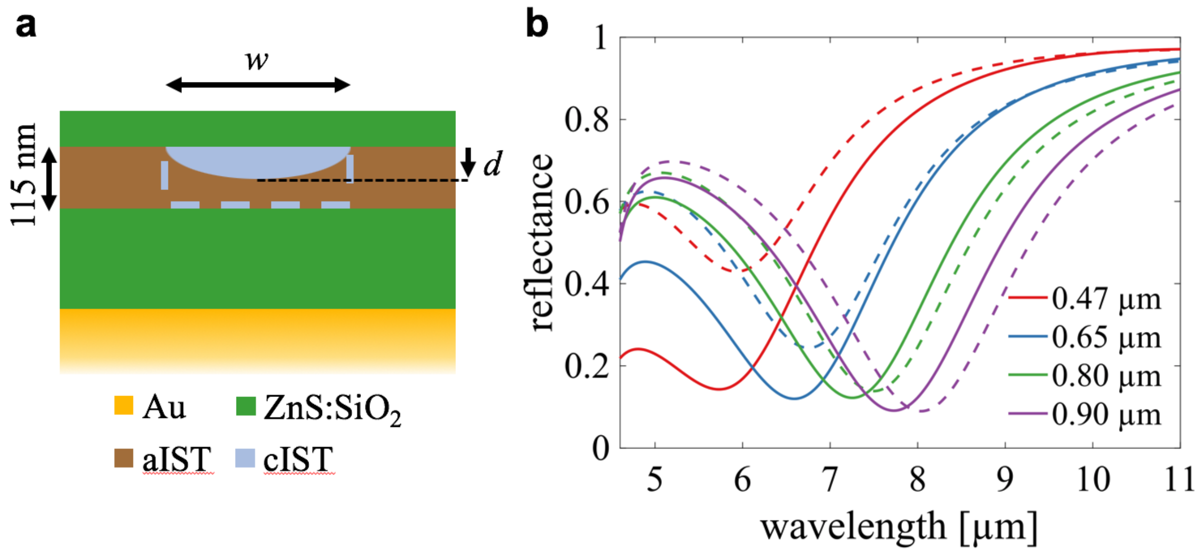

Supplementary Figure 11: Influence of the crystallization depth on performance in an IST-absorber. a) Sketch of the cross-section of the unit cell with a cIST grating bar of width *w* and crystallization depth *d*. The first approximation of a rectangular cross-section with full crystallization depth is sketched with dashed outlines **b)** Comparison of simulated spectra which account for smaller crystallization depths (solid) with those that do not (dashed). The angle of incidence is 20°. For the dashed lines, the cIST patch is simplified as a square brick with full crystallization depth d = 115 nm = *const*.

Supplementary Table 3: Laser pulse parameters for the cIST bars in Supplementary Figure 11. All bars were switched a pulse power of 0.4 mW and with 500 pulses during 1 second.

cIST bar width *w* [µm]	0.47	0.65	0.8	0.9
Pulse duration [ns]	250	310	340	370

Supplementary Note 10: Polarization-dependent infrared imaging with the IST absorber

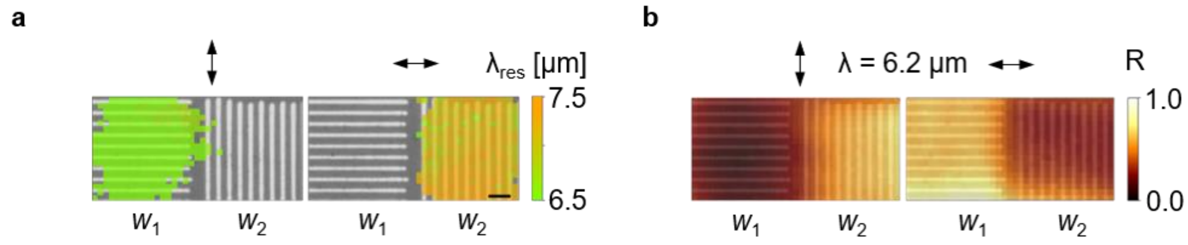

Supplementary Figure 12: a) Extracted data from hyperspectral infrared images for vertical (left) and horizontal (right) polarization: Color-coded resonance wavelengths of optically written cIST grating absorber detector pixels with two distinct design wavelengths (different bar widths *w*). Detector pixels with grating bars perpendicular to the incident light polarization (indicated by arrows above) absorb light (turned on), whereas detector pixels with bars parallel to the light polarization do not absorb light (turned off). Only the resonant areas (reflectance < 60%) are coloured. The scale bar is 4 µm. b) Infrared reflectance image of the grating absorbers from a) at 6.2 µm. Even though detector pixels are excited for a given light polarization, the reflectance is still highly wavelength selective: The not excited and off-resonance detector pixels appear bright, i.e. absorb little light, whereas the in-resonance detector pixels appear dark, i.e. absorb a lot of light. Same scale as for a)

To prove the viability of our design as a detector for infrared imaging where micro-bolometer detector pixel sizes in the ~20 µm range are state-of-the-art[16], we fabricated a checkerboard pattern composed of absorber pixels (1 detector pixel = 1 set of gratings) with two distinct design wavelengths (Supplementary Figures 12). By using cIST gratings with a period of 2 µm and bar widths w_1 = 680 nm (left grating) and w_2 = 790 nm (right grating) we created polarization-sensitive IR detector pixels. In general, one could also easily use cIST circular antenna arrays (or square net gratings) for polarization independent absorption. Light microscope images of the detector pixels are shown in the background. The pixels are only excited (i.e. absorb) if the incident light is polarized perpendicular to the bars. In Supplementary Figure 12a, the resonance wavelength is color-coded and resolved spatially. In the left image, light impinges with vertical polarization (indicated by the arrows above the image). Therefore, the left pixel is excited and absorbs light at λ_{res} = 6.5 µm (green), whereas the right pixel is not excited and simply reflects the light. For horizontal polarization of the incident light, however, the left pixel reflects the light and the right pixel absorbs light at λ_{res} = 7.5 µm (orange). How much light is absorbed by the excited pixels depends on how well their resonance wavelength matches the wavelength of the incident light. This spatially and spectrally selective, polarization-dependent operation is visualized in Supplementary Figure 12b. There, the spatially resolved, measured relative reflectance at a wavelength of 6.2 µm of the same pixels is shown again for both polarization directions. The unexcited pixels appear bright, i.e. absorb little light, whereas the excited pixels appear dark, i.e. absorb a lot of light. The absorptance reaches again ~90%. The excited left pixel appears darker than the excited right pixel because the operation wavelength of 6.2 µm is closer to the resonance wavelength of the left pixel. At another operation wavelength, the contrast would be different (see main text Figure 4b, d).

Supplementary Note 11: Nanoscale soldering of nanoantennas

As described in the main text, the presented resonance shift could be even larger if not for the shrinking of the Au antennas due to the annealing at 250°C in the oven. To monitor this effect, dimer antenna arrays of the same dimensions but without an IST patch in the dimer gaps were placed on the samples. In Supplementary Figure 13a, the transmittance spectra of these arrays are plotted before (solid lines) and after (dashed lines) the annealing. It can be seen that the annealing led to a significant blueshift of the resonances for all investigated antenna lengths. This is directly linked to the decreased antenna lengths. As a result, the resonance shift of the soldered together dimers is decreased accordingly in Figure 5e of the main text.
In an improvsed design, the Au antennas can be replaced with Al antennas which are covered by a very thin (3-5 nm) natural oxide. Additionally, the glass substrate from Figure 5d-f can be replaced with CaF_2 which has a much larger transparency window in the mid infrared. Consequently, the dimer antennas and thus the resonance shift can become much larger. In Supplementary Figure 13b, the resonance shift $\Delta\lambda_{res}$ of such dimer antennas is plotted against the antenna length l for different gap sizes g (see legend). It is evident that the shift becomes larger for increasing antenna lengths and increasing gap sizes. For realistic FWHM of about 800 nm (see Figure 5), this promises tuning figures of merit (TFOM) of between 1 and 4, and by extrapolation even larger, surpassing the "normal" TFOM $\lesssim$ 1.2 for plasmonic antennas with GeSbTe compounds[17] by far.

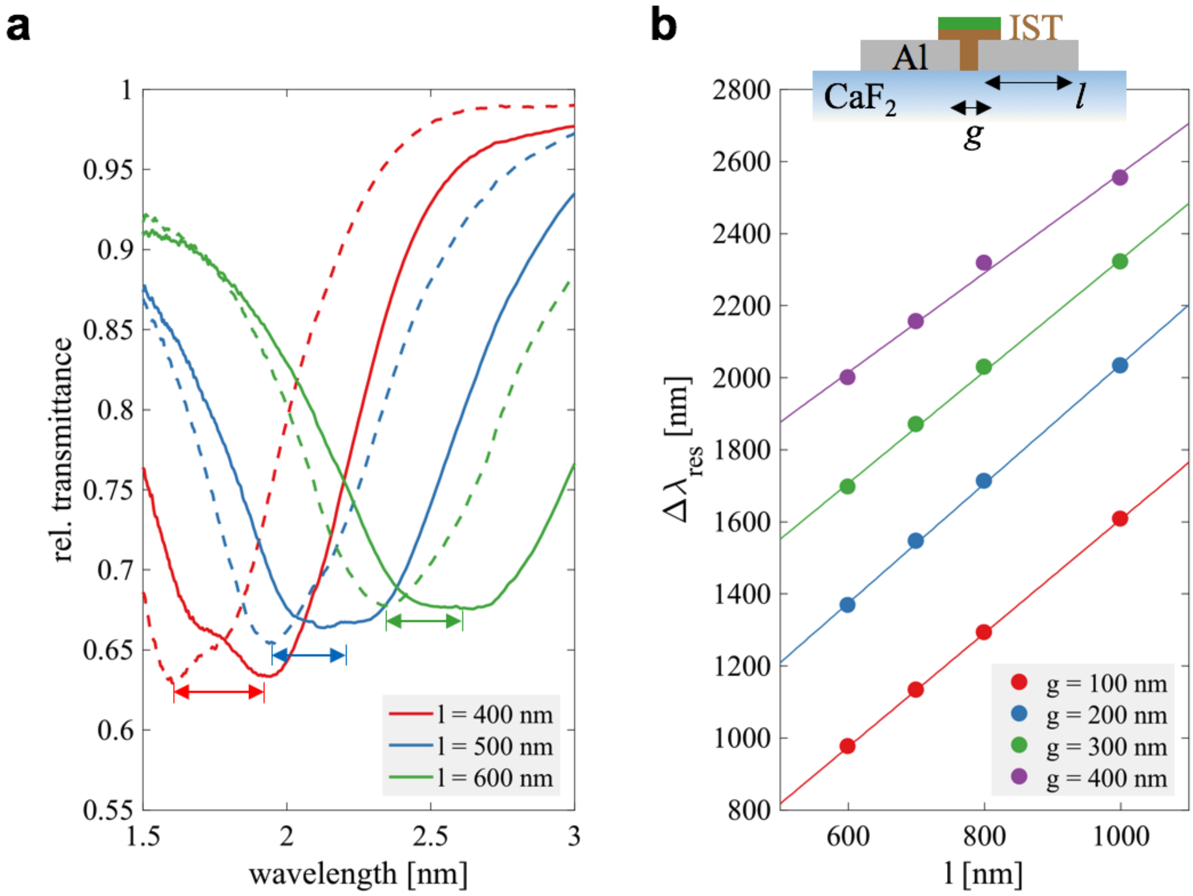

Supplementary Figure 13: Nanoscale soldering of dimer antennas. a) Measured transmittance spectra for the Au dimer antennas from Figure 5 (main text) without an IST patch before (solid) and after (dashed) annealing in the oven. A significant blue-shift by more than 300 nm after annealing is visible. This reduces the overall resonance shift observed for the IST-bridged dimers in Figure 5. **b)** Simulated resonance shift of IST-bridged Al-dimers on a CaF_2 substrate (see top inset). The resonance shift increases approximately linearly with the dimer gap size *g* and antenna length *l*.

Supplementary References

1. Bader, R. F. W. Atoms in molecules. *Acc. Chem. Res.* **18,** 9–15 (1985).
2. Gatti, C. Chemical bonding in crystals: new directions. *Z. Kristallogr. Cryst. Mater.* **220,** 399–457 (2005).
3. Bader, R. F. W. & Stephens, M. E. Spatial localization of the electronic pair and number distributions in molecules. *J. Am. Chem. Soc.* **97,** 7391–7399 (1975).
4. Fradera, X., Austen, M. A. & Bader, R. F. W. The Lewis Model and Beyond. *J. Phys. Chem. A* **103,** 304–314 (1999).
5. Kooi, B. J. & Wuttig, M. Chalcogenides by Design: Functionality through Metavalent Bonding and Confinement. *Adv. Mater.* **32,** 1908302 (2020).
6. Raty, J.-Y., Schumacher, M., Golub, P., Deringer, V. L., Gatti, C. & Wuttig, M. A Quantum-Mechanical Map for Bonding and Properties in Solids. *Adv. Mater.* **31,** 1806280 (2018).

7. Shportko, K., Kremers, S., Woda, M., Lencer, D., Robertson, J. & Wuttig, M. Resonant bonding in crystalline phase-change materials. *Nat. Mater.* **7,** 653–658 (2008).
8. Rausch, Pascal. Investigations of binary and ternary phase change alloys for future memory applications. *Dissertation, RWTH Aachen University* (2012).
9. Folland, T. G., Fali, A., White, S. T., Matson, J. R., Liu, S., Aghamiri, N. A., Edgar, J. H., Haglund, R. F., Abate, Y. & Caldwell, J. D. Reconfigurable infrared hyperbolic metasurfaces using phase change materials. *Nat. Commun.* **9,** 1–7 (2018).
10. Naik, G. V., Shalaev, V. M. & Boltasseva, A. Alternative Plasmonic Materials: Beyond Gold and Silver. *Adv. Mater.* **25,** 3264–3294 (2013).
11. Guler, U., Shalaev, V. M. & Boltasseva, A. Nanoparticle plasmonics: going practical with transition metal nitrides. *Materials Today* **18,** 227–237 (2015).
12. Ordal, M. A., Bell, R. J., Alexander, R. W., Long, L. L. & Querry, M. R. Optical properties of fourteen metals in the infrared and far infrared: Al, Co, Cu, Au, Fe, Pb, Mo, Ni, Pd, Pt, Ag, Ti, V, and W. *Appl. Opt., AO* **24,** 4493–4499 (1985).
13. Gholipour, B., Karvounis, A., Yin, J., Soci, C., MacDonald, K. F. & Zheludev, N. I. Phase-change-driven dielectric-plasmonic transitions in chalcogenide metasurfaces. *NPG Asia Mater.* **10,** 533–539 (2018).
14. Cubukcu, E. & Capasso, F. Optical nanorod antennas as dispersive one-dimensional Fabry–Pérot resonators for surface plasmons. *Appl. Phys. Lett.* **95,** 201101 (2009).
15. Michel, A.-K. U., Heßler, A., Meyer, S., Pries, J., Yu, Y., Kalix, T., Lewin, M., Hanss, J., Rose, A. D., Maß, T. W. W., Wuttig, M., Chigrin, D. N. & Taubner, T. Advanced Optical Programming of Individual Meta-Atoms Beyond the Effective Medium Approach. *Adv. Mater.* **31,** 1901033 (2019).
16. Tissot, J.-L. M., Tinnes, S., Durand, A., Minassian, C., Robert, P., Vilain, M. & Yon, J.-J. High-performance uncooled amorphous silicon video graphics array and extended graphics array infrared focal plane arrays with 17-µm pixel pitch. *Opt. Eng.* **50,** 061006 (2011).
17. Michel, A.-K. U., Wuttig, M. & Taubner, T. Design Parameters for Phase-Change Materials for Nanostructure Resonance Tuning. *Adv. Opt. Mater.* **5,** 1700261 (2017).
